# Assessing the impact of pneumococcal conjugate vaccines on invasive pneumococcal disease using polymerase chain reaction-based surveillance: an experience from South Africa

**DOI:** 10.1186/s12879-015-1198-z

**Published:** 2015-10-26

**Authors:** Stefano Tempia, Nicole Wolter, Cheryl Cohen, Sibongile Walaza, Claire von Mollendorf, Adam L. Cohen, Jocelyn Moyes, Linda de Gouveia, Susan Nzenze, Florette Treurnicht, Marietjie Venter, Michelle J. Groome, Shabir A. Madhi, Anne von Gottberg

**Affiliations:** Influenza Division, Centers for Disease Control and Prevention, Atlanta, Georgia USA; Influenza Program, Centers for Disease Control and Prevention, Pretoria, South Africa; Centre for Respiratory Diseases and Meningitis, National Institute for Communicable Diseases of the National Health Laboratory Service, Johannesburg, South Africa; School of Pathology, Faculty of Health Sciences, University of the Witwatersrand, Johannesburg, South Africa; School of Public Health, Faculty of Health Sciences, University of the Witwatersrand, Johannesburg, South Africa; Medical Research Council, Respiratory and Meningeal Pathogens Research Unit, University of the Witwatersrand, Johannesburg, South Africa; Division of Global Health Protection, Centers for Disease Control and Prevention, Pretoria, South Africa; Department of Science and Technology/National Research Foundation: Vaccine Preventable Diseases, University of the Witwatersrand, Johannesburg, South Africa

**Keywords:** Pneumococcus, Conjugate vaccine, *lytA*, Molecular serotyping, South Africa

## Abstract

**Background:**

The use of molecular diagnostic techniques for the evaluation of the impact of pneumococcal conjugate vaccines (PCVs) has not been documented. We aimed to evaluate the impact of PCVs on invasive pneumococcal disease (IPD) using polymerase chain reaction (PCR)-based techniques and compare with results obtained from culture-based methods.

**Methods:**

We implemented two independent surveillance programs for IPD among individuals hospitalized at one large surveillance site in Soweto, South Africa during 2009–2012: (i) PCR-based (targeting the *lytA* gene) syndromic pneumonia surveillance; and (ii) culture-based laboratory surveillance. Positive samples were serotyped. The molecular serotyping assay included targets for 42 serotypes including all serotypes/serogroups included in the 7-valent (PCV-7) and 13-valent (PCV-13) PCV. The Quellung reaction was used for serotyping of culture-positive cases. We calculated the change in rates of IPD (*lytA*- or culture-positive) among HIV-uninfected children aged <2 years from the year of PCV-7 introduction (2009) to the post-vaccine years (2011 or 2012).

**Results:**

During the study period there were 607 *lytA*-positive and 1,197 culture-positive cases that were serotyped. Samples with *lytA* cycle threshold (Ct)-values ≥35 (30.2 %; 123/407) were significantly less likely to have a serotype/serogroup detected for serotypes included in the molecular serotyping assay than those with Ct-values <35 (78.0 %; 156/200) (*p* < 0.001). From 2009 to 2012 rates of PCV-7 serotypes/serogroups decreased −63.8 % (95 % CI: −79.3 % to −39.1 %) among *lytA*-positive cases and −91.7 % (95 % CI: −98.8 % to −73.6 %) among culture-positive cases. Rates of *lytA*-positive non-vaccine serotypes/serogroups also significantly decreased (−71.7 %; 95 % CI: −81.1 % to −58.5 %) over the same period. Such decline was not observed among the culture-positive non-vaccine serotypes (1.2 %; 95 % CI: −96.7 % to 58.4 %).

**Conclusions:**

Significant downward trends in IPD PCV-7 serotype-associated rates were observed among patients tested by PCR or culture methods; however trends of non-vaccine serotypes/serogroups differed between the two groups. Misclassifications of serotypes/serogroups, affecting the use of non-vaccine serotypes as a control group, may have occurred due to the low performance of the serotyping assay among *lytA*-positive cases with high Ct-values. Until PCR methods improve further, culture methods should continue to be used to monitor the effects of PCV vaccination programs on IPD incidence.

**Electronic supplementary material:**

The online version of this article (doi:10.1186/s12879-015-1198-z) contains supplementary material, which is available to authorized users.

## Background

Every year pneumococcal disease results in ≈ 600,000 deaths among children <5 years of age globally, with the majority of deaths occurring in Africa [1]. While over 90 *Streptococcus pneumoniae* serotypes have been identified [2], approximately 20 are responsible for the majority of invasive pneumococcal disease (IPD) [3]. The direct and indirect effects of the pneumococcal conjugate vaccines (PCVs), which target the most common serotypes associated with IPD, have been documented in several high-income countries [4–7].

In 2009, South Africa introduced the 7-valent PCV (PCV-7) into its routine infant immunization program using a schedule of vaccination at 6 and 14 weeks and a booster dose at 9 months [4]. PCV-7 was replaced by the 13-valent PCV (PCV-13) in April 2011 [4]. The benefit of the introduction of PCV-7 and subsequently PCV-13 have been documented in South Africa using data from a nationwide, laboratory-based IPD surveillance program [4]. IPD cases were detected through the identification of *S. pneumoniae* from cultured specimens that were subsequently serotyped using the Quellung reaction [5].

The determination of pneumococcal serotypes is key to assess the effects of PCVs, including decreases in PCV serotypes and potential non-PCV serotype replacement following the use of the vaccine over time. With PCVs being progressively introduced into the routine infant immunization programs of several low- and middle-income countries [6], serotype-specific pneumococcal surveillance is key to assess the impact of the vaccine in diverse settings.

Culture remains the gold standard for the identification of the organism while the Quellung reaction remains the gold standard for serotype determination from available isolates. Nonetheless, culture, while highly specific, has low sensitivity, requires long incubation periods and is not commonly available in many low-income countries [7]. In addition, antibiotic therapy prior to specimen collection or suboptimal culturing conditions may reduce the yield of cultures [8, 9].

Polymerase chain reaction (PCR)-based methods targeting pneumococcal specific genes, such as *lytA*, have resulted in improved and timely diagnosis of pneumococcal diseases [10–12]. Such methods can be easily implemented where molecular diagnostic capacity exists and could become an alternative diagnostic tool in settings where culture capacity is lacking or suboptimal. Nonetheless, the use of molecular diagnostic techniques for the evaluation of the impact of PCVs against IPD has not been documented.

We aimed to evaluate the impact of PCVs on IPD using PCR-based methods at one large surveillance site in South Africa from 2009 through 2012, and compare these results with those obtained from culture-based methods.

## Methods

### Description of the surveillance programs

#### The Severe Acute Respiratory Illness (SARI) program (PCR-based syndromic surveillance)

We conducted active, prospective, syndromic, hospital-based surveillance at Chris Hani-Baragwanath Academic Hospital (CHBAH) from February 2009 through December 2012. This hospital is the only public hospital serving a well-defined community (Soweto, Gauteng Province) of about 1.4 million people in 2012 [13] from which rates of hospitalizations can be estimated [14, 15]. We aimed to test all enrolled individuals with *lytA* real-time PCR on whole blood. For the SARI program a case of bacteremic pneumococcal pneumonia (BPP) was defined as the identification of *S. pneumoniae* in blood specimens using a single-target (*lytA*) quantitative real-time PCR assay adapted from Carvalho et al. [16]. *lytA*-positive specimens (cycle threshold (Ct)-value < 40) were serotyped by real-time PCR using an adaption of the method described by Azzari *et al.* [17]. The molecular serotyping assay included targets for 42 serotypes including all serotypes/serogroups included in PCV-7 and PCV-13 PCV (see Supplementary Material for Additional file 1). DNA extraction was performed using the Roche MagNA Pure LC 1.0 instrument during May 2009-January 2010, the Roche MagNA Pure LC 2.0 instrument during February 2010-July 2012 and the Roche MagNA Pure 96 instrument during August-December 2012.

#### The Group for Enteric, Respiratory and Meningeal Disease Surveillance (GERMS) program (culture-based laboratory surveillance)

Data on active, laboratory-based IPD surveillance conducted under the GERMS program at CHBAH were included in this study. For the GERMS program, IPD cases were defined as hospitalized persons from whom *S. pneumoniae* was cultured from specimens that are normally sterile (e.g., cerebrospinal fluid (CSF), blood or joint fluid). Cultures were taken as clinically indicated by attending clinical staff. Strains were serotyped by the Quellung reaction targeting 93 serotypes [5].

**Fig. 1:**
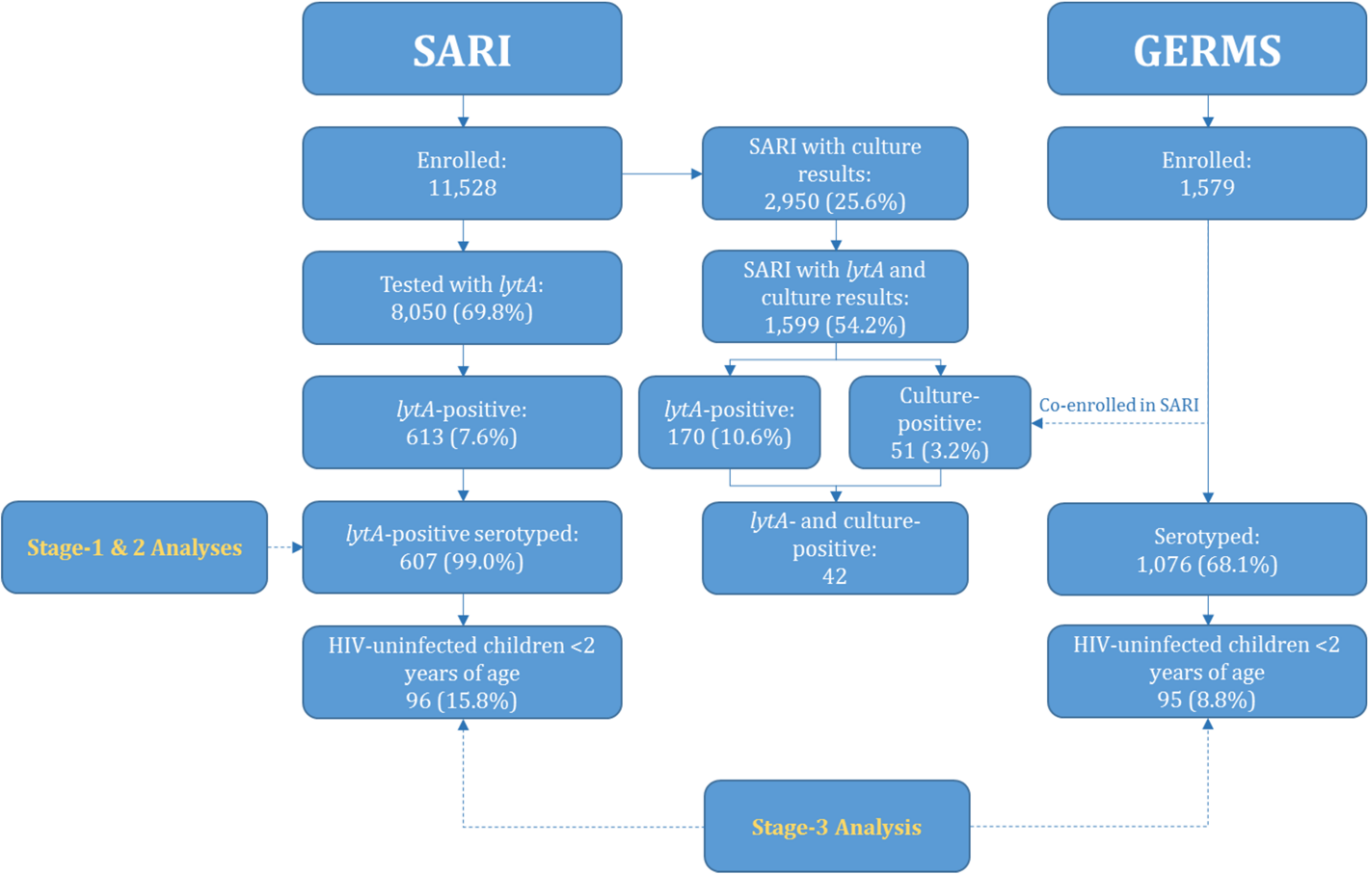
Enrolment of cases with severe acute respiratory illness (SARI program) and cases of culture-positive invasive pneumococcal disease (GERMS program) hospitalized at Chris Hani-Baragwanath Academic Hospital, Soweto, South Africa, 2009–2012

The study and laboratory procedures of the SARI and GERMS programs have been previously described [4, 14, 15, 18] and are summarized in Additional file 1. While the SARI and GERMS surveillance programs were implemented independently, co-enrolment of patients was possible. This would have been in instances when a patient tested positive for *S. pneumoniae* on culture locally, but also meet the SARI case definition. In addition, while the GERMS program only enrolled patients with *S. pneumoniae*-positive culture results, results of any blood culture (including negative results) taken routinely on-site were collected under the SARI program. These culture were taken as clinically indicated by attending clinical staff. Fig. 1 provides the enrolment of cases under the SARI and GERMS programs, including co-enrolment.

Written informed consent was obtained from all cases who were 18 years of age and older. Proxy informed consent was obtained from parents or legal guardians of minors.

### Statistical analysis

We implemented a 3-stage analysis whereby stage-1 and −2 analyses were conducted to inform the interpretation of results of the main analysis in stage 3. The analytical approach for each analysis is described in Additional file 1. The analysis was implemented using Stata 13.1 (StataCorp®, Texas, USA).

#### Stage-1 analysis: proportion of serotypable samples by* lytA* Ct-value among *lytA*-positive patients with SARI

In the stage-1 analysis we evaluated the proportion of serotypable samples by *lytA* Ct-value among the *lytA*-positive SARI samples obtained from patients of any age (Fig. 1). We conducted this analysis because we suspected that *lytA*-positive samples with high Ct-values would be associated with a low performance of the molecular serotyping assay as previously reported [19].

#### Stage-2 analysis: factors associated with increasing Ct-values among *lytA*-positive patients with SARI

In the stage-2 analysis we evaluated factors associated with increasing Ct-values among *lytA*-positive SARI patients of any age (Fig. 1). We conducted this analysis because in the stage-1 analysis we observed a low performance of the serotyping assay for *lytA*-positive samples with high Ct-values. Variable performance of the serotyping assay could impact the interpretation of the trend analysis of BPP cases by vaccine serotypes (stage-3 analysis). In particular, a variation in the proportion of *lytA*-positive samples with higher or lower Ct-values over time could result in varying proportions of serotypable samples affecting the observed trends of BPP by vaccine serotype.

#### Stage-3 analysis: time-trends of BPP (*lytA*-positive) and IPD (culture-positive) among HIV-uninfected children <2 years of age

The aim of the study was to assess the feasibility of evaluating the impact of PCVs using PCR-based methods, and therefore, for the main analysis we focused on HIV-uninfected children <2 years of age (Fig. 1). This group was chosen because it is directly vaccinated and the effectiveness of PCV has been well documented in several countries [20–23], including South Africa [4].

To assess the trends of BPP over time we calculated the annual rate of *lytA*-positive SARI hospitalizations overall and by PCV-7, additional PCV-13 and non-vaccine serotypes/serogroups during 2009–2012. We assessed the impact of the introduction of PCV-7 and PCV-13 by calculating the reduction in rates of BPP (expressed as percentage reduction with associated 95 % confidence intervals) between the post-vaccine years (2011 or 2012) and the year of introduction of PCV (2009). A similar trend analysis was implemented using the culture-positive cases. Rates were expressed per 100,000 person-years.

### Ethical approval

The SARI protocol was approved by the University of the Witwatersrand Human Research Ethics Committee (M081042) and the University of KwaZulu-Natal Biomedical Research Ethics Committee (BF157/08). The GERMS protocol was approved by the research ethics committee of the University of the Witwatersrand (M081117).

## Results

### Description of SARI cases

From February 2009 through December 2012, 8,050/11,528 (69.8 %) of SARI cases enrolled at CHBAH were tested for whole blood *lytA*, of which 613 (7.6 %) were *lytA*-positive (Fig. 1). The *lytA*-detection rate varied by age: 4.9 % (130/2639), 5.5 % (24/438), 8.3 % (44/530), 9.5 % (269/2841), 10.2 % (135/1321) and 2.7 % (7/258) among individuals <2, 2–4, 5–24, 25–44, 45–64 and ≥65 years of age, respectively (p < 0.001). The *lytA*-detection rate varied also by year: 8.0 % (129/1616) in 2009, 7.5 % (173/2293) in 2010, 6.3 % (152/2421) in 2011 and 9.2 % (159/1720) in 2012 (*p* = 0.005). In 2012, the *lytA*-detection rate was higher among samples from which DNA was extracted using the Roche MagNA Pure 96 instrument (14.8 %; 73/492) than using the Roche MagNA Pure LC 2.0 instrument (7.0 %; 86/1228) (*p* < 0.001).

Of the 613* lytA*-positive cases, 607 (99.0 %) were tested with the serotyping assay and were included for further analyses. The HIV serostatus was known for 558/607 (91.9 %) individuals of which 395 (70.8 %) were HIV positive. The HIV prevalence varied by age: 11.9 % (13/109), 20.0 % (4/20), 82.1 % (32/39), 94.1 % (240/255), 81.1 % (103/127) and 33.3 % (2/6) among individuals <2, 2–4, 5–24, 25–44, 45–64 and ≥65 years of age, respectively (*p* < 0.001).

A culture result was available for 2,950/11,528 (25.6 %) SARI cases, of which 69 (2.3 %) tested positive for *S. pneumoniae*. Among the 1599 SARI cases with both *lytA* and culture results available, 179 (11.2 %) tested positive in at least one of the assays. Of these, 170 (95.0 %) and 51 (28.5 %) specimens tested positive for *lytA* and culture, respectively; 128 (71.5 %) cases tested positive for *lytA* alone, 9 (5.0 %) for culture alone and 42 (23.5 %) for both *lytA* and culture. The detection rate was 10.6 % (170/1599) and 3.2 % (51/1599) for *lytA* and culture, respectively (*p* < 0.001).

Among the 607/613 (99.0 %) *lytA*-positive samples that were tested with the serotyping assay, 166 (27.3 %) had available culture results and 42 (25.3 %) tested positive for *S. pneumoniae*; 16/29 (55.2 %), 11/33 (33.3 %) and 15/104 (14.4 %) among samples with *lytA* Ct-value of ≤30, 31–34 and ≥35, respectively (p < 0.001). Among the 42 cases that tested positive in both assays, a serotype could be identified in 36 (85.7 %) cases; 32 (76.2 %) cases using the Quellung reaction and 26 (61.9 %) cases using the molecular serotyping assay. A serotype could be identified by both assays in 22/36 (61.1 %) cases. Among these, the same serotype/serogroup was identified by both assays in 21 (95.5 %) cases. A serotype could be identified by the Quellung reaction, but not by the molecular serotyping assay in 10/36 (27.8 %) cases. Of these, 8 (80.0 %) were serotypes/serogroups included in the molecular serotyping assay, of which 7 (87.5 %) had a *lytA* Ct-value ≥35 and 1 (12.5 %) had a lytA Ct-value of 34. All of them were PCV-7, PCV-13 or 6A serotypes. A serotype/serogroup could be identified by the molecular serotyping assay, but not by the Quellung reaction in 4/36 (11.1 %) cases. The characteristics of the 36 cases for which a serotype/serogroup was identified are provided in Table 1.

**Table 1:** Characteristics of *S. pneumoniae*-positive cases (*N* = 36) hospitalized at Chris Hani Baragwanath Academic Hospital for which a serotype/serogroup could be identified by the Quellung reaction and/or the molecular serotyping assay, Soweto, South Africa, 2009-2012^a^

Age group (in years)	HIV serostatus	*lytA* Ct-value	Serotype/serogroup	
			Quellung reaction	Molecular serotyping assay
*lytA* Ct-value ≤30				
25–44	Pos	25	19 F	19B/19 F
45–64	Pos	26	1	1
<2	Pos	27	10A^c^	Neg42
25–44	Pos	27	19 F	19B/F
25–44	Unknown	27	Not available^b^	18A/B/C
25–44	Pos	27	19A	19A
25–44	Pos	28	19A	19A
25–44	Pos	28	19 F	19B/F
25–44	Pos	29	19A	19A
25–44	Pos	29	19A	19A
25–44	Pos	30	19A	19A
*lytA* Ct-value 31–34				
25–44	Pos	31	3	3
45–64	Unknown	31	19A	19A
25–44	Pos	31	1	1
25–44	Pos	32	12 F	12A/B/F
25–44	Neg	32	19A	19A
45–64	Pos	33	4	4
25–44	Pos	33	Not available^b^	19A
25–44	Neg	34	1	1
25–44	Pos	34	Not available^b^	1
25–44	Pos	34	1^d^	Neg42
45–64	Pos	34	19A	19A
*lytA* Ct-value ≥35				
5–24	Pos	35	9 V	9A/L/N/V
<2	Pos	35	23 F	23 F
5–24	Pos	35	19A^d^	Neg42
25–44	Pos	35	1^d^	Neg42
<2	Neg	36	6A	6A/B
<2	Unknown	36	6B	6A/B
5–24	Pos	36	18C^d^	Neg42
25–44	Pos	37	19A^d^	Neg42
2–4	Neg	37	14^d^	Neg42
25–44	Pos	37	Not available^b^	1
25–44	Pos	38	1^d^	Neg42
25–44	Pos	38	23A^c^	Neg42
2–4	Pos	39	6A^d^	Neg42
<2	Neg	39	19A^e^	18A/B/C^e^

Abbreviations: HIV: human immunodeficiency virus; Ct-value: cycle threshold value; Neg42: samples that tested negative for the 42 serotypes detected by the serotyping assay

^a^ Discrepant or missing serotype/serogroup results are in bolt font

^b^ Isolate not available for serotyping using the Quellung reaction

^c^ Serotype not included in the molecular serotyping assay

^d^ Serotype included in the molecular serotyping assay

### Stage-1 analysis: proportion of serotypable samples by *lytA* Ct-value among *lytA*-positive patients with SARI

**Fig. 2:**
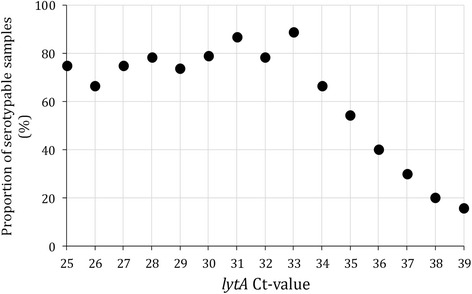
Proportion of serotypable *lytA*-positive samples (*n* = 607) by *lytA* cycle threshold value (Ct-value) among patients hospitalized with severe acute respiratory illness at Chris Hani-Baragwanath Academic Hospital, Soweto, South Africa, 2009–2012. Serotypable samples were samples tested with the serotyping assay from which a serotype/serogroup included in the assay was detected

Of the 607 *lytA*-positive SARI samples that were tested with the serotyping assay, a serotype/serogroup included in the assay was detected in 279 (46.0 %) samples. Among these, the most frequently detected serotypes/serogroups were 19A (61; 21.8 %), 1 (52; 18.6 %) and 6A/B (33; 11.8 %). The *lytA* Ct-value ranged between 25 and 39 (median 36). We observed a decline of the proportion of serotypable samples among samples with an individual *lytA* Ct-value ≥34 (Fig. 2 and Table 2). However, compared to samples with Ct-values ≤30 this decline was statistically significant among samples with individual Ct-values ≥35 (Table 2). The proportion of serotypable samples declined from 76.1 % (54/71) among samples with Ct-value ≤30 to 15.7 % (8/51) among samples with Ct-value of 39 (p < 0.001). Overall, the proportion of samples with Ct-value <34 or <35 was 26.0 % (158/607) and 32.9 % (200/607), respectively.

**Table 2:** Proportion of serotypable^a^ *lytA*-positive samples (*n* = 607) by *lytA* cycle threshold value (Ct-value) among patients hospitalized with severe acute respiratory illness at Chris Hani-Baragwanath Academic Hospital, Soweto, South Africa, 2009–2012

*lytA* Ct-value	Serotypable^a^ *lytA*-positive samples		
	n/N (%)	OR (95 % CI)	*p*
≤30	54/71 (76.1)	Reference	-
31	20/23 (86.9)	2.1 (0.6-7.9)	0.275
32	22/28 (78.6)	1.2 (0.4-3.3)	0.790
33	32/36 (88.9)	2.5 (0.8-8.1)	0.123
34	28/42 (66.7)	0.6 (0.3-1.5)	0.281
35	31/57 (54.4)	0.4 (0.2-0.8)	0.011
36	29/72 (40.3)	0.2 (0.1-0.4)	<0.001
37	28/93 (30.1)	0.1 (0.07-0.3)	<0.001
38	27/134 (20.1)	0.08 (0.04-0.15)	<0.001
39	8/51 (15.7)	0.06 (0.02-0.14)	<0.001

Abbreviations: OR: odds ratio; CI: confidence interval

^a^Serotypable samples were samples tested with the serotyping assay from which a serotype/serogroup included in the assay was detected

### Stage-2 analysis: factors associated with increasing Ct-values among *lytA*-positive patients with SARI

Among the 607 *lytA*-positive SARI samples that were tested with the serotyping assay, 71 (11.7 %) had Ct-values ≤30, 129 (21.2 %) had Ct-values 31–34 and 407 (67.1 %) had Ct-values ≥35. On multivariable analysis (Table 3), factors negatively associated with increasing *lytA* Ct-values were: (i) extraction instrument Roche MagNA Pure LC 2.0 (adjusted odds ratio [aOR]: 0.4; 95 % confidence intervals [CI]: 0.2-0.6) or Roche MagNA Pure 96 (aOR: 0.3; 95 % CI: 0.1-0.7) compared to Roche MagNA Pure LC 1.0; (ii) HIV infection (aOR: 0.4; 95 % CI: 0.2-0.7); (iii) duration of hospitalization for 3–7 days (aOR: 0.4; 95 % CI: 0.2-0.8) or ≥8 days (aOR: 0.3; 95 % CI: 0.1-0.5) compared to 0–2 days; and (iv) in-hospital death (aOR: 0.3; 95 % CI: 0.2-0.6). Additional PCV-13 serotypes/serogroups were significantly less associated with increasing *lytA* Ct-values (aOR: 0.3; 95 % CI: 0.2-0.5), while non-vaccine serotypes/serogroups were significantly more associated with increasing *lytA* Ct-values (aOR: 2.7; 95 % CI: 1.6-4.6) compared with PCV-7 serotypes/serogroups (Table 3).

**Table 3:** Factors associated with increasing *lytA* cycle threshold value (Ct-value) among *lytA*-positive patients hospitalized with severe acute respiratory illness at Chris Hani-Baragwanath Academic Hospital, Soweto, South Africa, 2009–2012

Variable	*lytA* Ct-value				Proportional-Odds Model			
					Univariate analysis		Multivariable analysis	
	Total n (%)	≤30 n (%)	31-34 n (%)	≥35 n (%)	OR^b^ (95 % CI)	*p*-value	aOR^b^ (95 % CI)	*p*-value
Age (in years)	*N* = 603	*N* = 71	*N* = 128	*N* = 404				
<2	125 (20.7)	6 (8.5)	11 (8.6)	108 (26.7)	Reference	-		
2–4	24 (4.0)	0 (0.0)	6 (4.7)	18 (4.5)	0.5 (0.2-1.5)	0.227		
5–24	44 (7.3)	2 (2.8)	13 (10.2)	29 (7.2)	0.3 (0.2-0.7)	0.008		
25–44	268 (44.4)	48 (67.6)	59 (46.1)	161 (39.8)	0.2 (0.1-0.4)	<0.001		
45–64	135 (22.4)	14 (19.7)	37 (28.9)	84 (20.8)	0.3 (0.1-0.5)	<0.001		
≥65	7 (1.2)	1 (1.4)	2 (1.5)	4 (1.0)	0.2 (0.1-1.1)	<0.051		
Sex	*N* = 603	*N* = 71	*N* = 128	*N* = 404				
Male	257 (42.6)	27 (38.0)	64 (50.0)	166 (41.1)	Reference	-		
Female	346 (57.4)	44 (62.0)	64 (50.0)	238 (58.9)	1.1	0.453		
Year	*N* = 607	*N* = 71	*N* = 129	*N* = 407				
2009	129 (21.3)	8 (11.3)	18 (13.9)	103 (25.3)	Reference	-		
2010	173 (28.5)	34 (47.9)	46 (35.7)	93 (22.8)	0.3 (0.2–0.5)	<0.001		
2011	150 (24.7)	10 (14.1)	39 (30.2)	101 (24.8)	0.6 (0.3–0.9)	0.033		
2012	155 (25.5	19 (26.8)	26 (20.2)	110 (27.0)	0.6 (0.3–1.1)	0.064		
Extraction Instrument	*N* = 607	*N* = 71	*N* = 129	*N* = 407				
Roche MagNA Pure LC 1.0	136 (22.4)	9 (12.7)	19 (14.7)	108 (26.5)	Reference	-	Reference	-
Roche MagNA Pure LC 2.0	400 (65.9)	54 (76.1)	96 (74.4)	250 (61.4)	0.4 (0.3–0.7)	<0.001	0.4 (0.2–0.6)	<0.001
Roche MagNA Pure 96	71 (11.7)	8 (11.3)	14 (10.8)	49 (12.0)	0.6 (0.3–1.1)	0.092	0.3 (0.1–0.7)	0.004
Antibiotics 24H before admission	*N* = 601	*N* = 71	*N* = 128	*N* = 402				
No	567 (94.3)	68 (95.8)	122 (95.3)	377 (93.8)	Reference	-		
Yes	34 (5.7)	3 (4.2)	6 (4.7)	25 (6.2)	1.4 (0.6–3.0)	0.393		
Antibiotics during admission	*N* = 586	*N* = 69	*N* = 126	*N* = 391				
No	19 (3.2)	3 (4.3)	2 (1.6)	14 (3.6)	Reference	-		
Yes	567 (96.8)	66 (95.6)	124 (98.4)	377 (96.4)	0.8 (0.3–2.2)	0.647		
Underlying medical conditions^a^	*N* = 603	*N* = 71	*N* = 128	*N* = 404				
No	565 (93.7)	66 (93.0)	120 (93.7)	379 (93.8)	Reference	-		
Yes	38 (6.3)	5 (7.0)	8 (6.3)	25 (6.2)	0.9 (0.5–1.8)	0.839		
HIV infection	N = 558	N = 66	N = 119	N = 373				
No	163 (29.2)	5 (7.6)	22 (18.5)	136 (36.5)	Reference	-	Reference	-
Yes	395 (70.8)	61 (92.4)	97 (81.5)	237 (63.5)	0.3 (0.2–0.5)	<0.001	0.4 (0.2–0.7)	0.001
PCV serotypes/serogroups	*N* = 607	*N* = 71	*N* = 129	*N* = 407				
PCV-7	111 (18.3)	13 (18.3)	28 (21.7)	70 (17.2)	Reference	-	Reference	-
PCV-13	138 (22.7)	35 (49.3)	62 (48.1)	41 (10.1)	0.3 (0.2–0.5)	<0.001	0.3 (0.2–0.5)	<0.001
NVT	358 (59.0)	23 (32.4)	39 (30.2)	296 (72.7)	2.7 (1.7–4.4)	<0.001	2.7 (1.6–4.6)	<0.001
Duration of symptoms (in days)	*N* = 602	*N* = 71	*N* = 127	*N* = 404				
0–2	204 (33.9)	14 (19.7)	38 (29.9)	152 (37.6)	Reference	-		
≥3	398 (66.1)	57 (80.3)	89 (70.1)	252 (62.4)	0.6 (0.4–0.8)	0.003		
Duration of hospitalization (in days)	*N* = 602	*N* = 71	*N* = 129	*N* = 402				
0–2	92 (15.3)	1 (1.4)	15 (11.6)	76 918.9)	Reference	-	Reference	-
3–7	267 (44.3)	26 (36.6)	61 (47.3)	180 (44.8)	0.4 (0.2–0.8)	0.004	0.5 (0.2–1.1)	0.071
≥8	243 (40.4)	44 (62.0)	53 (41.1)	146 (36.3)	0.3 (0.1–0.5)	<0.001	0.3 (0.1–0.6)	0.002
In-hospital outcome	*N* = 603	*N* = 71	*N* = 129	*N* = 403				
Survived	562 (93.2)	58 (81.7)	119 (92.2)	385 (95.5)	Reference	-	Reference	-
Died	41 (6.8)	13 (18.31)	10 (7.8)	18 (4.5)	0.3 (0.2–0.6)	<0.001	0.3 (0.2–0.7)	0.003

Abbreviations: OR: odds ratio; aOR: adjusted odds ratio; CI: confidence interval; HIV: human immunideficency virus; PCV-7: 7-valent pneumococcal conjugate vaccine serotypes (included serotypes/serogroups 4, 6A/B, 9A/V/L/N, 14, 18A/B/C, 19B/F, 23 F); PCV-13: additional 13-valent pneumococcal conjugate vaccineserotypes (included serotypes/serogroups 1, 3, 5, 7A/F, 19A); NVT: serotypes/serogroups not included in PCV-7 or PCV-13, including samples that tested negative for the 42 serotypes detected by the serotyping assay

^a^ Underlying medical conditions included: asthma, chronic lung disease, chronic heart disease, liver disease, renal disease, diabetes mellitus, immunocompromizing conditions excluding HIV infection or neurological disease

^b^ The odds ratio of the proportional-odds model measures the effect of a predictor on the odds of being above a specified level, compared with the odds of being at or below the specified level

### Stage-3 analysis: time-trends of BPP (*lytA*-positive) and IPD (culture-positive) among HIV-uninfected children <2 years of age

**Fig. 3:**
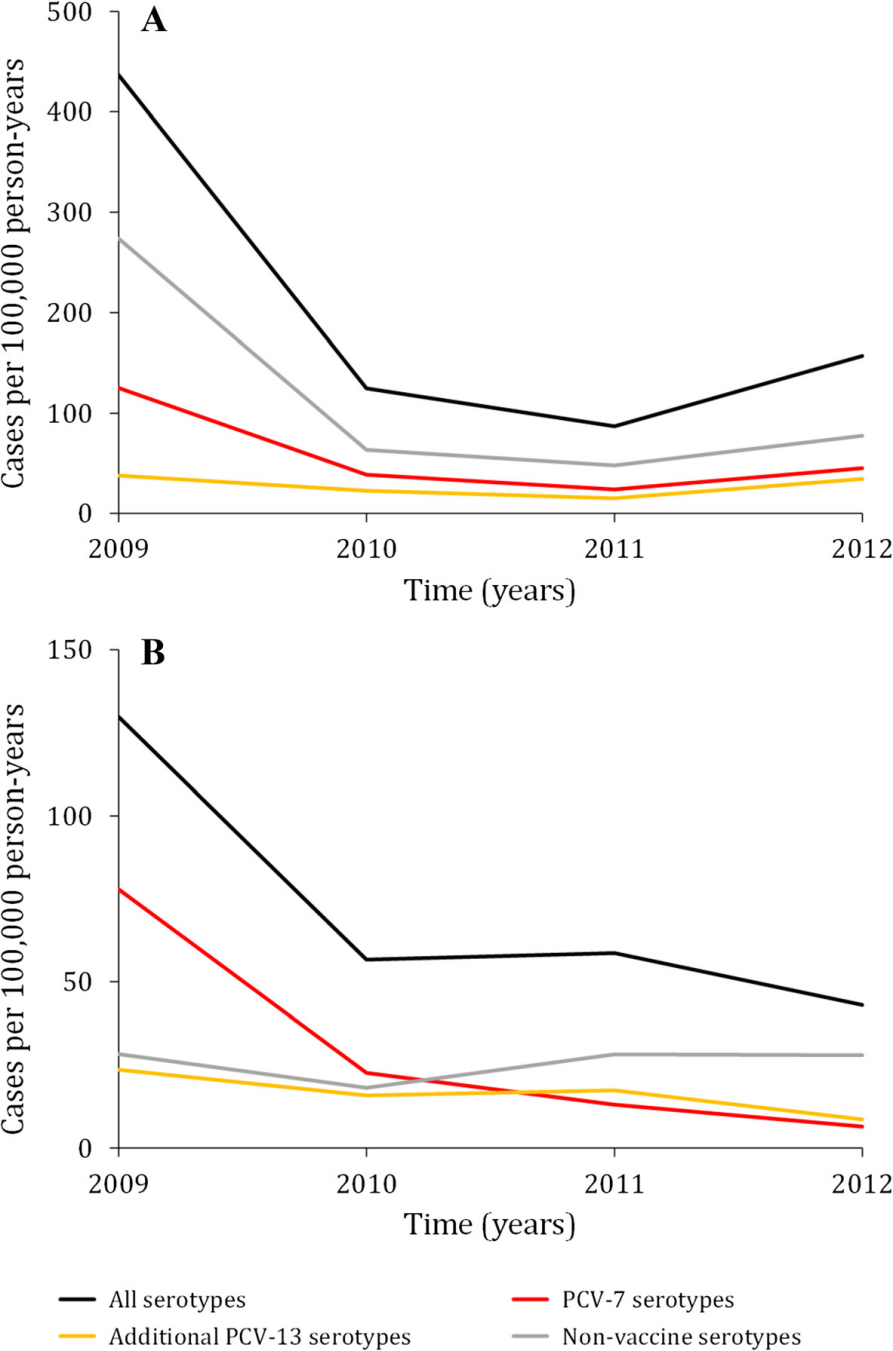
Rates of invasive *S. pneumoniae*-associated hospitalizations among HIV-uninfected children <2 years of age at Chris-Hani Baragwanath Academic Hospital, Soweto, South Africa, 2009–2012. **a**: *lytA*-positive cases (SARI program) (7-valent pneumococcal conjugate vaccine (PCV-7) serotypes/serogroups included: 4, 6A/B, 9A/V/L/N, 14, 18A/B/C, 19B/F, 23 F; additional 13-valent pneumococcal conjugate vaccine (PCV-13) serotypes/serogroups included: 1, 3, 5, 7A/F, 19A). **b**: culture-positive cases (GERMS program) (7-valent pneumococcal conjugate vaccine (PCV-7) serotypes included: 4, 6A/B, 9 V, 14, 18C, 19 F, 23 F; additional 13-valent pneumococcal conjugate vaccine (PCV-13) serotypes included: 1, 3, 5, 7 F, 19A). Non-vaccine serotypes included serotypes/serogroups not included in PCV-7 or PCV-13, including samples that tested negative for the 42 serotypes detected by the serotyping assay for *lytA*-positive samples

The proportion of PCV serotypes/serogroups among *lytA*-positive (SARI) and culture-positive (GERMS) cases is provided in Additional file 1 (Table S1 and Figure S1). Overall from 2009 to 2012, among HIV-uninfected children <2 years of age a reduction in rates of −64.0 % (95 % CI: −72.9 % to −52.6 %) was observed among *lytA*-positive cases compared to −66.8 % (95 % CI: −81.2 % to −43.8 %) among culture-positive cases (Table 4). Over the same period, rates of PCV-7 serotypes/serogroups decreased −63.8 % (95 % CI: −79.3 % to −39.1 %) among *lytA*-positive cases and −91.7 % (95 % CI: −98.4 % to −73.6 %) among culture-positive cases. Rates of *lytA*-positive non-vaccine serotypes/serogroups also significantly decreased (−71.7 %; 95 % CI: −81.1 % to −58.5 %) over the same period. Such decline was not observed among the culture-positive non-vaccine serotypes (1.2 %; 95 % CI: −96.7 % to 58.4 %). Among *lytA*-positive cases the time-trends of non-vaccine serotypes/serogroups mimicked closely those of PCV-7 serotypes/serogroups and the rates of non-vaccine serotypes/serogroups were consistently higher than those of PCV-7 and PCV-13 serotypes/serogroups even during the year of vaccine introduction (2009) (Fig. 3a). This was not observed for culture-positive cases (Fig. 3b).

**Table 4:** Rates of bacteremic pneumococcal pneumonia (SARI program – *lytA*-positive) and invasive pneumococcal pneumonia (GERMS program – culture-positive) among HIV-uninfected children <2 years of age hospitalized at Chris Hani-Baragwanath Academic Hospital, Soweto, South Africa, 2009–2012.

PCV serotypes	Hospitalization rates per 100,000 person-years			Relative difference in hospitalization rates			
	2009	2011	2012	2009 to 2011		2009 to 2012	
	Rate (95 % CI)	Rate (95 % CI)	Rate (95 % CI)	% (95 % CI)	*p*	% (95 % CI)	*p*
Any *lytA*-positive (SARI program)							
PCV-7	125.1 (93.7–163.6)	23.9 (11.9–42.8)	45.2 (28.0–69.1)	−80.9 (−90.9 to −62.9)	<0.001	−63.8 (−79.3 to −39.1)	<0.001
PCV-13	37.8 (21.6–61.3)	15.2 (6.1–31.3)	34.4 (19.7–55.9)	−59.7 (−85.9 to +3.4)	0.067	+8.8 (−94.8 to +57.3)	0.796
NVT	273.7 (226.2–328.3)	47.8 (30.0–72.4)	77.5 (54.3–107.3)	− 82.5 (−89.4 to −72.3)	<0.001	−71.7 (−81.1 to −58.5)	<0.001
All	436.6 (375.9–504.2)	86.9 (62.1–118.3)	157.1 (123.2–197.6)	−80.1 (−86.2 to −71.8)	<0.001	−64.0 (−72.9 to −52.6)	<0.001
Culture-positive (GERMS program)							
PCV-7	77.9 (53.6–109.3)	13.0 (4.8–28.4)	6.5 (1.3–18.9)	−83.2 (−94.2 to – 59.5)	<0.001	−91.7 (−98.4 to −73.6)	<0.001
PCV-13	23.6 (11.3–43.4)	17.4 (7.5–34.2)	8.6 (2.3–22.1)	−26.3 (−74.7 to +107.3)	0.529	−63.5 (−91.6 to +26.5)	0.084
NVT	28.3 (14.6–49.5)	28.2 (15.0–48.3)	28.0 (14.9–47.8)	−0.2 (−58.0 to +139.2)	0.993	+1.2 (−96.7 to +58.4)	0.974
All	129.8 (97.8–168.9)	58.6 (38.7–85.3)	43.1 (26.3–66.5)	−54.8 (−72.6 to −27.1)	<0.001	−66.8 (−81.2 to −43.8)	<0.001

Abbreviations: PCV-7: 7-valent pneumococcal conjugate vaccine serotypes (included serotypes/serogroups 4, 6A/B, 9A/V/L/N, 14, 18A/B/C, 19B/F, 23 F for *lyA*-positive samples and 4, 6A/B, 9 V, 14, 18C, 19 F, 23 F for culture-positive samples); PCV-13: additional 13-valent pneumococcal conjugate vaccine serotypes (included serotypes/serogroups 1, 3, 5, 7A/F, 19A for *lyA*-positive samples and 1, 3, 5, 7 F, 19A for culture-positive samples); NVT: serotypes/serogroups not included in PCV-7 or PCV-13, including samples that tested negative for the 42 serotypes detected by the serotyping assay for *lytA*-positive samples

An increase in rates of *lytA*-positive cases was observed from 2011 to 2012 for all PCV categories (Table 4 and Fig. 3a), while this was not observed among culture-positive cases for which declines in PCV-7 and PCV-13 were observed (Table 4 and Fig. 3b).

From 2009 to 2011, the time-trends and the proportional decrease in rates of PCV-7 serotypes/serogroups was similar among *lytA*-positive (−80.9 %; 95 % CI: −90.9 % to −62.9 %) and culture-positive (−83.2 %; 95 % CI: −94.2 % to −59.5 %) cases (Table 4 and Fig. 3a and b). The sharpest decline of PCV-7 serotypes/serogroups was observed from 2009 to 2010 for both *lytA*- (Fig. 3a) and culture-positive cases (Fig. 3b). Among *lytA*-positive cases a sharper decline was observed between 2009 and 2010 for non-vaccine serotypes/serogroups (−76.8 %; 95 % CI: −81.3 % to −69.2 %) compared to PCV-7 serotypes/serogroups (−53.2 %; 95 % CI: −64.7 % to −41.6 %).

## Discussion

We expected that the introduction of PCV into our national immunization program would lead to declines in pneumococcal disease, especially vaccine-type disease among the vaccinated population. This has been shown from surveillance data using traditional culture-based methods [4, 20–23], and the expectation was that this would also be seen in surveillance using newer molecular techniques. Overall, using both PCR- and culture-based methods we reported a significant decline of BPP or IPD rates during the early years of PCV-7 introduction among HIV-uninfected children <2 years of age in Soweto. Nonetheless, the PCR-based results would have been difficult to interpret in the absence of culture-based data because molecular methods showed a decline in vaccine-type as well as non-vaccine-type disease and laboratory testing results were sensitive to the bacterial load and equipment used.

As expected and previously reported [4], rates of *lytA*- and culture-positive PCV-7 serotypes/serogroups significantly declined over the study period (stage-3 analysis), probably owing to the progressive effect of the introduction of PCV-7 into the routine infant immunization program. Nevertheless, rates of *lytA*-positive non-vaccine serotypes, which should not be impacted by the use of PCV-7, unexpectedly also significantly decreased and were consistently higher than those of PCV-7 and PCV-13 over the study period. Consistent with previous studies [20–23], including data from South Africa [4], this was not observed for culture-positive cases.

In the stage-1 analysis, we observed a significant reduction of the proportion of serotypable samples (i.e., positive for one of the serotypes/serogroups detected by the serotyping assay) among *lytA*-positive cases with Ct-values ≥35 as previously reported [19]. The low performance of the serotyping assay among *lytA*-positive cases with high Ct-values could result in the misclassification of the PCV serotypes/serogroups as non-vaccine types (non-vaccine serotypes/serogroups including samples negative for the 42 serotypes detected by the serotyping assay: Neg42) as observed among cases with available serotype results from both molecular- and culture-based methods. This could potentially explain the high rates and the downward trends observed in the non-vaccine serotype group (stage-3 analysis). The significant positive association of non-vaccine compared to PCV-7 serotypes/serogroups with increasing Ct-values (stage-2 analysis) increases the plausibility of this hypothesis. In addition, the fact that the time-trends of non-vaccine serotypes/serogroups among any *lytA*-positive cases (Ct-value <40) mimicked closely those of PCV-7 serotypes/serogroups (stage-3 analysis) further suggests that, while *lytA*-positive samples with Ct-values ≥35 could not be accurately serotyped and hence were classified as Neg42, they were probably true cases that included misclassified PCV serotypes/serogroups.

Of note is that the proportion of vaccine and non-vaccine serotypes/serogroups was similar among culture-positive and *lytA*-positive cases with Ct-value <35 (Additional file1). This further suggests that more reliable molecular serotype results can be obtained from samples with *lytA* Ct-values <35 as observed in the stage-1 analysis. In addition, it appears that non-vaccine serotypes potentially included in the PCV-7 (9A/L/N, 18A/B and 19B) and PCV-13 (7A) categories (as a result of potential misclassification of non-vaccine serotypes as vaccine serotypes within serogroups) did not significantly alter the proportion of vaccine and non-vaccine categories compared to serotype-specific culture results. The non-vaccine serotypes potentially misclassified as vaccine types in this study accounted for <1 % of the overall burden of IPD in previous studies conducted in South Africa [4].

Nonetheless, in our study only ≈ 33 % of all *lytA*-positive samples had a Ct-value <35, hindering our ability to implement a trend analysis using more conservative Ct-value cut-offs, especially when focusing on specific age and HIV-serostatus groups. While the overall number of *lytA*-positive cases obtained in our study was well above the number of cases needed to significantly estimate a decline in PCV serotypes using population based methods [24], this was not the case when restricting the analysis to *lytA*-positive cases with Ct-value <35.

A significantly higher decline of non-vaccine compared to PCV-7 serotypes/serogroups was observed from 2009 to 2010 potentially owing to the combined effect of the reduction of PCV-7 serotypes/serogroups misclassified in the non-vaccine category as well as the use of the Roche MagNA Pure LC 2.0 extraction instrument from February 2010. In the stage-2 analysis the use of the Roche MagNA Pure LC 2.0 compared to the Roche MagNA Pure LC 1.0 instrument for DNA extraction was significantly less associated with increasing *lytA* Ct-values. This suggests that the use of a better extraction instrument would increase the proportion of *lytA*-positive samples with low Ct-values and consequently increase their likelihood to be correctly serotyped using the serotyping assay (stage-1 analysis). This would result in improved classifications of PCV-7 and PCV-13 serotype/serogroups (increasing rates in these categories) and consequently reduced misclassification of the same serotype/serogroups (decreasing rates in the non-vaccine category) in 2010 compared to 2009. The replacement of the MagNA Pure LC 2.0 instrument with the Roche MagNA Pure 96 instrument in August 2012 could also have introduced bias in the trend analysis. In 2012, the detection rate of *lytA*-positive cases doubled following the introduction of the new instrument potentially resulting in the rate increase observed from 2011 to 2012, whereas this was not observed among culture-positive cases. This highlights the importance of standardization of procedures over time for time-trend analysis purposes. Nonetheless, while the standardization of methods across the study period is key to avoid the introduction of biases, this may conflict with the use of rapidly evolving technology and the need to upgrade laboratory equipment over time.

In the stage-2 analysis, besides the use of different extraction instruments and the non-vaccine serotypes/serogroups, factors negatively associated with increasing Ct-values were HIV infection and in-hospital death. The *lytA* Ct-value provides a semi-quantitative measure of the pneumococcal load, with lower Ct-values indicative of higher load and vice-versa. The association of high pneumococcal load among *lytA*-positive cases with HIV infection and in-hospital deaths has been previously reported [18].

Among *lytA*-positive samples with available culture results the proportion of culture-positive samples decreased with increasing *lytA* Ct-values, and was only ≈ 55 % even among *lytA*-positive samples with Ct-values ≤30. This highlights the usefulness of the use of PCR-based methods for improved diagnosis of pneumococcal disease as previously reported [10–12].

Our study has limitations that warrant discussion. First, we did not have *lytA* data for years prior to the introduction of PCV-7 and our data were limited to one large surveillance site in the country. Nonetheless, data from nationwide culture-based surveillance reported downward trends of IPD PCV-7-associated rates [4] similar to those reported in this analysis. Second, we did not systematically test all enrolled patients using PCR- and culture-based methods hindering our ability to directly compare results from the same group of patients. Nonetheless, the proportion of vaccine and non-vaccine type disease was similar between syndromes (i.e., meningitis, bacteremic pneumonia and bacteremia without focus) among South African children <5 years of age during the pre-vaccine era [25] and there was no statistically significant difference in the proportion of vaccine- and non-vaccine-type disease between blood- and CSF-positive specimens among HIV-uninfected children <2 years of age in this study. Last, the molecular serotyping assay targets only 42 serotypes/serogroups leaving uncertainty about the *lytA-*positive samples that tested negative for the 42 targets.

## Conclusions

In conclusion, in our setting the overall downward trends in IPD PCV-7 serotypes-associated rates were similar among patients tested with PCR- or culture-based methods; however trends of non-vaccine serotypes/serogroups differed between the two groups. While PCR-based methods could be used to assess trends of PCV-7 serotypes/serogroups the misclassifications observed in this study affected the use of non-vaccine types as a control group. Such misclassifications could also potentially hinder the ability to assess serotype replacement following the use of PCVs over time. These findings suggest that current molecular methods alone may not be sufficient to monitor the impact of PCV unless standardized procedures and equipment are used throughout the study period and large populations are systematically surveyed to allow time-trend analysis using more restrictive Ct-value cut-offs. If the results of this study are confirmed in other settings, the development of improved molecular serotyping assays would enhance serotype-specific pneumococcal surveillance using PCR-based methods. Improvements of the molecular serotyping assays would entail increased sensitivity and inclusion of targets for all serotypes/serogroups.

### Ethics

The SARI protocol was approved by the University of the Witwatersrand Human Research Ethics Committee (M081042) and the University of KwaZulu-Natal Biomedical Research Ethics Committee (BF157/08). The GERMS protocol was approved by the research ethics committee of the University of Witwatersrand and by local hospitals or provincial ethics committees as required.

## Additional file

Additional file 1: **Assessing the Impact of Pneumococcal Conjugate Vaccines on Invasive Pneumococcal Disease Using Polymerase Chain Reaction-Based Surveillance: An Experience from South Africa (Supplementary Material).** (DOCX 65 kb)

## Abbreviations

BPP: Bacteremic Pneumococcal Pneumonia
CHBAH: Chris Hani-Baragwanath Academic Hospital
CSF: Cerebrospinal Fluid
Ct-value: Cycle Threshold Value
GERMS: Group for Enteric, Respiratory and Meningeal Disease Surveillance
IPD: Invasive Pneumococcal Diseases
PCR: Polymerase Chain Reaction
PCV: Pneumococcal Conjugate Vaccine
PCV-13: 13-Valent Pneumococcal Conjugate Vaccine
PCV-7: 7-Valent Pneumococcal Conjugate Vaccine
*S. pneumoniae*: *Streptococcus pneumoniae*
SARI: Severe Acute Respiratory Illness
