# Supplementary material for: Assessing the impact of pneumococcal conjugate vaccines on invasive pneumococcal disease using polymerase chain reaction-based surveillance: an experience from South Africa
Source: BMC Infect Dis. 2015 Oct 26;15:450. doi: 10.1186/s12879-015-1198-z (PMC4620746; doi:10.1186/s12879-015-1198-z)
Supplement: Supplementary file 1 — Assessing the Impact of Pneumococcal Conjugate Vaccines on Invasive Pneumococcal Disease Using Polymerase Chain Reaction-Based Surveillance: An Experience from South Africa (Supplementary Material). (DOCX 65 kb) [file 12879_2015_1198_MOESM1_ESM.docx]

**Assessing the Impact of Pneumococcal Conjugate Vaccines on Invasive Pneumococcal Disease Using Polymerase Chain Reaction-Based Surveillance: An Experience from South Africa (Supplementary Material)**

**Authors**

Stefano Tempia^1,2,3^, Nicole Wolter^3,4^, Cheryl Cohen^3,5^, Sibongile Walaza^3^, Claire von Mollendorf^3^, Adam L. Cohen^1,2^, Jocelyn Moyes^3,5^, Linda de Gouveia^3^, Susan Nzenze^5,6^, Florette Treurnicht^3^, Marietjie Venter^6^, Michelle J. Groome^7,8^, Shabir A. Madhi^3,7,8^, Anne von Gottberg^3,4^

**Affiliations**

^1^Influenza Division, Centers for Disease Control and Prevention, Atlanta, Georgia, United States of America

^2^Influenza Program, Centers for Disease Control and Prevention, Pretoria, South Africa

^3^Centre for Respiratory Diseases and Meningitis, National Institute for Communicable Diseases of the National Health Laboratory Service, Johannesburg, South Africa

^4^School of Pathology, Faculty of Health Sciences, University of the Witwatersrand, Johannesburg, South Africa

^5^School of Public Health, Faculty of Health Sciences, University of the Witwatersrand, Johannesburg, South Africa

^6^Division of Global Health Protection, Centers for Disease Control and Prevention, Pretoria, South Africa

^7^Respiratory and Meningeal Pathogens Research Unit, University of the Witwatersrand, Johannesburg, South Africa

^8^Department of Science and Technology/National Research Foundation: Vaccine Preventable Diseases, University of the Witwatersrand, Johannesburg, South Africa

**METHODS**

**Description of the surveillance programs**

*The Severe Acute Respiratory Illness (SARI) program*

Patients that presented at Chris Hani Baragwanath Academic Hospital (CHBAH) from Monday to Friday meeting the severe acute respiratory illness (SARI) case definition and with symptom onset within 7 days from admission were eligible for enrollment, except for adult patients where enrolment occurred for two of every five working days per week due to large patient numbers (enrolment days varied according to the intake days of the two participating wards). Numbers of patients admitted, numbers meeting the study case definitions and numbers enrolled were collected throughout the study period.

A case of SARI was defined as a hospitalized person who had illness onset within 7 days of admission and who met age-specific clinical inclusion criteria. A case in children aged 2 days to <3 months included any hospitalized patient with diagnosis of suspected sepsis or physician-diagnosed acute lower respiratory tract infection irrespective of signs and symptoms. A case in children aged 3 months to <5 years included any hospitalized patient with physician-diagnosed acute lower respiratory tract infection, including bronchitis, bronchiolitis, pneumonia and pleural effusion. A case in individuals aged ≥5 years included any hospitalized patient presenting with manifestation of acute lower respiratory tract infection with fever (>38°C) or history of fever and cough or sore throat and shortness of breath or difficult breathing with or without clinical or radiographic findings of pneumonia.

Study staff completed case report forms until discharge and collected nasopharyngeal (NP) aspirates from children <5 years of age and nasopharyngeal swabs from individuals ≥5 years of age as well as blood specimens. All decisions on medical care, including requests for cultures, were undertaken at the discretion of the attending physician. When available, culture results performed at site were recorded. Data in case report forms were reviewed regularly to identify inconsistencies and regular site visits were conducted to ensure adherence to study procedures.

*The Group for Enteric, Respiratory and Meningeal Disease Surveillance (GERMS) program*

In 2012, over 200 laboratories representing more than 450 hospitals actively participated in the GERMS program. Patients with invasive pneumococcal disease (IPD) were defined as hospitalized persons from whom *S. pneumoniae* was cultured from specimens that are normally sterile (e.g., cerebrospinal fluid, blood or joint fluid). Reports of laboratory-confirmed IPD together with isolates and demographic details (e.g. age, sex, date of specimen collection and body fluid source of isolate) were recorded from all hospitals. Enhanced surveillance at 24 sentinel sites (including CHBAH) collected additional information including HIV serostatus of enrolled patients.

**Detection and serotyping of *S. pneumoniae***

All blood samples and IPD isolates/clinical specimens collected from CHBAH under the SARI and GERMS surveillance programs were processed at the Centre for Respiratory Diseases and Meningitis (CRDM), National Institute for Communicable Diseases (NICD) of the National Health laboratory Services (NHLS), Johannesburg, South Africa. The CRDM bacteriology laboratory is a SANAS (South African National Accreditation System) accredited laboratory since 2008.

*Samples collected under the Severe Acute Respiratory Illness (SARI) program*

For the SARI program, collection and testing of blood samples at CHBAH started in May 2009. Whole blood samples were collected within 24 hours of hospital admission. Specimens were stored at 4°C at site and transported to the CRDM for testing. DNA was extracted from 200 μl of whole blood using the DNA Isolation kit III for bacteria for the Roche MagNA Pure LC 1.0 or 2.0 instruments and the MagNA Pure 96 DNA and viral NA SV kit for the Roche MagNA Pure 96 instrument according to the manufacturer’s instructions. Extracted DNA was eluted into 100 μl of elution buffer and stored at -20°C.

A case of bacteremic pneumococcal pneumonia (BPP) was defined as the identification of *S. pneumoniae* in the blood specimen using a single-target (*lytA*) quantitative real-time PCR assay adapted from Carvalho *et al.* [[[1]](#endnote-1)]. *lytA*-positive specimens (cycle threshold (Ct) value<40) were serotyped by real-time PCR using an adaption of the method described by Azzari *et al.* [[[2]](#endnote-2)]. Reactions were performed as duplex reactions and detected serotypes/serogroups 1, 3, 4, 5, 6A/B, 6C/D, 7A/F, 8, 9A/L/V/N, 10A/B, 12A/B/F, 14, 15A/B/C/F, 18A/B/C, 19A, 19B/F, 20, 22A/F/38, 23F, 33A/F/37, 35B and 38. The serotyping assay included targets for all serotypes/serogroups included in PCV-7 or PCV-13. Altogether, taking into account the mixed serotypes or serogroups the assay detected 42 serotypes. If a specimen tested negative (Ct-value ≥40) in all eleven reactions, it was considered to be a serotype not included in the assay (Neg42) and consequentially not a PCV-7 or PCV-13 serotype.

For the interpretation of the results of this analysis, it should be noted that molecular serotyping assays, like those used in this study, have intrinsic limitations. Mainly, the high genotypic similarities between the capsular loci of certain serotypes make it difficult to develop a serotype-specific assay, and, therefore, certain serogroups remain unresolved to specific serotypes [[[3]](#endnote-3)]. If serogroups including vaccine serotypes are included in the relevant PCV category, like in our study, then the proportional contribution of the non-vaccine serotypes to the overall burden of pneumococcal disease would be lower and those of vaccine serotypes would be higher than the true value. Nonetheless, this would not affect trends as it is expected that non-vaccine serotypes would not be affected by the introduction of PCV during the early years of introduction when serotype replacement is not expected; hence trends of vaccine groups would be driven by changes among the vaccine serotypes even if non-vaccine serotypes are included in the PCV categories. However, even if the introduction of PCV in the routine infant immunization program would result in the elimination of all PCV serotypes a “base rate” of PCV serotype will remain, owing to the potential inclusion of non-vaccine serotypes in the PCV categories. In addition, limited serotypes are included in the serotype assays to reduce the time and labor required, and, therefore, not all serotypes are detected, including potentially important non-vaccine serotypes. This would hinder the ability to detect possible serotype replacement.

*Samples collected under the Group for Enteric, Respiratory and Meningeal Disease Surveillance (GERMS) program*

*S. pneumoniae* isolates or clinical specimens (in the event than an organism could not be cultured) obtained from the GERMS program were submitted to the CRDM for testing. Upon receipt of specimens, the bacterial strains were subcultured from Dorset transport medium onto 5% horse blood Columbia base agar (Oxoid, Hampshire, UK). The culture identification was confirmed using standard microbiological procedures [[[4]](#endnote-4)]. Strains were serotyped by the Quellung reaction [[[5]](#endnote-5)] using type-specific antisera (Statens Serum Institute, Copenhagen, Denmark).

**Determination of HIV status**

For the SARI program HIV results were obtained from a combination of two sources: (i) patient clinical records when available and (ii) for consenting patients, an anonymized linked dried blood spot was tested at NICD. When both results were available, the NICD result was used. For the GERMS program HIV results performed at the participating sites were obtained from patient clinical records only. Testing included HIV enzyme-linked immunosorbent assay (ELISA) testing for patients ≥18 months and PCR testing for children <18 months.

**Definitions**

For this study underlying medical conditions included; chronic lung disease, chronic heart disease, liver disease, renal disease, diabetes mellitus, immunocompromizing conditions excluding HIV infection or neurological disease. Comorbidities were considered absent in cases for which the medical records stated that the patient had no underlying medical condition or when there was no direct reference to that condition.

*lytA*-positive (SARI) specimen serotypes were categorized as PCV-7 serotypes/serogroups (4, 6A/B, 9A/V/L/N, 14, 18A/B/C, 19B/F, 23F), additional PCV-13 serotypes/serogroups not included in PCV-7 (1, 3, 5, 7A/F, 19A), and non-vaccine serotypes (all serotypes/serogroups not included in PCV-7 or PCV-13, including samples classified as Neg42). Because the serotype assay cannot differentiate serotypes within certain serogroups we included serogroups that included a vaccine serotype in the corresponding PCV category. This resulted in the potential inclusion of non-vaccine serotypes in a specific vaccine group (e.g., 6A, 9A/L/N, 18A/B and 19B in the PCV-7 group; and 7A in the PCV-13 group) and the exclusion of the same serotype from the non-vaccine group.

Culture-positive (GERMS) specimen serotypes were categorized as PCV-7 serotypes (4, 6A/B, 9V, 14, 18C, 19F, 23F; 6A was included in this group because of known cross-protection [[[6]](#endnote-6)]), additional PCV-13 serotypes not included in PCV-7 (1, 3, 5, 7F, 19A), and non-vaccine serotypes (all serotypes not included in PCV-7, PCV-13 or 6A).

**Statistical analysis**

*Stage-1 analysis: proportion of serotypable samples by lytA Ct-value among lytA-positive patients with SARI*

For this analysis we used logistic regression to compare the proportion of serotypable samples with *lytA* Ct-values ≤30 to the proportion of serotypable samples with individual *lytA* Ct-values from 31-39. A sample was considered serotypable if one of the serotypes/serogroups included in the molecular serotyping assay could be detected.

*Stage-2 analysis: factors associated with increasing Ct-values among lytA-positive patients with SARI*

For this analysis we used a proportional-odds (ordinal) model, which assumes that the ordinal outcome variable represents categories of an underlying continuous variable. The outcome variable (*lytA* Ct-value) was categorized in 3 levels: ≤30, 31-34 and ≥35. The coefficients of the proportional-odds model (and associated odds ratio [OR]) measure the effect of a predictor on the log odds of being above a specified level, compared with the log odds of being at or below the specified level. For the multivariable model we assessed all variables that were significant at p<0.2 on univariate analysis, and dropped non-significant factors (p≥0.05) with stepwise backward selection. Patients with missing data for included variables were dropped from the model.

*Stage-3 analysis: time-trends of bacteremic pneumococcal pneumonia (lytA-positive) and invasive pneumococcal disease (culture-positive) among HIV-uninfected children <2 years of age*

We estimated the rate of *lytA*-positive BPP hospitalizations (SARI program) among HIV-uninfected children aged <2 years per 100,000 person-years using the number of BPP hospitalizations, adjusting for non-enrollment (refusal to participate and non-enrollment during weekends [[[7]](#endnote-7),[[8]](#endnote-8)]) for each year using the following formula:

$BPP_{Rate_{i,j}}=\frac{SARI_{Enrolled_{i}}*\frac{7}{5}*\frac{1}{X_{i}}*lytA_{DetectRate_{i,j}}*HIV_{PerNeg_{i,j}}}{Pop_{i}}*100,000$ (1)

Where $BPP_{Rate_{i,j}}$ is the estimated rate of BPP hospitalization among HIV-uninfected children aged <2 years associated with vaccine serotypes category *j* (PCV-7, additional PCV-13 and non-vaccine serotypes) in year *i* (2009-2012); $SARI_{Enrolled_{i}}$is the number of SARI cases aged <2 years enrolled in year *i*; 7/5 is the coefficient used to adjust for non-enrolment over weekends; *X_i_* is the proportion of all eligible children aged <2 years that were enrolled in year *i* (obtained from study logs); $lytA_{DetectRate_{i,j}}$is the detection rate for vaccine serotypes category *j* among SARI cases aged <2 years tested by *lytA* PCR in year *i*; $HIV_{PerNeg_{i,j}}$is the proportion of HIV-uninfected SARI cases among children aged <2 years with known HIV serostatus in year *i* and vaccine serotype category *j*; and *Pop_i_* is the population at risk (e.g. HIV-uninfected children aged <2 years in Soweto) in year *i*.

Since under the SARI program collection of blood samples started in May 2009, we imputed the expected number of cases from January through April using the monthly average of cases enrolled from May through December.

For this analysis we assumed that the prevalence of vaccine serotype categories and HIV infection was the same among individuals tested and not tested. Age- and year specific population denominators for Soweto were obtained from Statistics South Africa [[[9]](#endnote-9)], while age- and year-specific HIV prevalence in the study population was obtained from the projections of the Actuarial Society of South Africa AIDS and Demographic model [[[10]](#endnote-10)].

A similar trend analysis was implemented using the culture-positive cases. However, this analysis was limited to enrolled cases since from the GERMS program the proportion of non-enrollment was not available for adjustment.

**RESULTS**

**Proportion of pneumococcal conjugate vaccine (PCV) serotypes/serogroups among *lytA*-positive (SARI) and culture-positive (GERMS) cases**

Of the 607 *lytA*-positive SARI cases that were tested with the serotyping assay, age was known for 603 (99.3%) cases. Of these, 355 (58.9%) were non-vaccine serotypes/serogroups; 70.4% (88/125) and 55.9% (267/478) among individuals <2 and ≥2 years of age, respectively (Table S1). Among the 199/603 (33.0%) *lytA*-positive cases with Ct-values <35, 31.1% (62) were non-vaccine serotypes/serogroups; 41.2% (7/17) and 30.2% (55/182) among individuals <2 and ≥2 years of age, respectively (Table S1). The proportion of samples with *lytA* Ct-values <35 was lower among children <2 years of age (13.6%; 17/125) than among individuals aged ≥2 years (38.1%; 182/478) (p<0.001). The HIV prevalence among children <2 years with known HIV status was 11.9% (13/109).

Of the 1,579 culture-positive IPD cases enrolled, 1,076 (68.1%) tested positive from blood, 411 (26.0%) from cerebro-spinal fluid (CSF), 62 (3.9%) from pleural fluid and 30 (2.0%) from other specimens. Serotype results were available for 1,197 (75.8%) specimens. Of these 374 (31.2%) were non-vaccine serotypes; 33.7% (62/184) and 30.8% (312/1013) among individuals <2 and ≥2 years of age, respectively (Table S1). The HIV prevalence among children <2 years with known HIV status and available serotype results was 39.5% (62/157). The proportion of non-vaccine serotypes was significantly higher among *lytA*-positive samples with Ct-values <40 compared to culture-positive samples (58.9%; vs. 31.2%; p<0.001). This was not observed among *lytA*-positive samples with Ct-values <35 (31.1% vs. 31.2%; p=0.977). Of the 95 culture-positive HIV-uninfected children <2 years of age with available serotype results, 59 (62.1%) tested positive from blood and 36 (37.9%) from CSF. There was no significant difference in the proportion of non-vaccine serotypes between blood- (20/59; 33.9%) and CSF-positive (13/36; 36.1%) specimens (p=0.975). The time-trends of culture-positive cases by specimen type are provided in Figure S1.

**Table S1:** Proportion of pneumococcal conjugate vaccine (PCV) serotypes/serogroups among *lytA*-positive (SARI) and culture-positive (GERMS) cases hospitalized at Chris Hani Baragwanath Academic Hospital, Soweto, South Africa, 2009-2012.

| **PCV serotypes** | **Year** | | | | |
| --- | --- | --- | --- | --- | --- |
|  | **2009**  **n (%)** | **2010**  **n (%)** | **2011**  **n (%)** | **2012**  **n (%)** | **Total**  **n (%)** |
| **Any *lytA*-positive case (SARI program)** | | | | | |
| **<2 year of age** | **N=36** | **N=21** | **N=33** | **N=35** | **N=125** |
| PCV-7 | 12 (33.3) | 4 (19.1) | 4 (12.1) | 4 (11.4) | 24 (19.2) |
| PCV-13 | 4 (11.1) | 2 (9.5) | 4 (12.1) | 3 (8.6) | 13 (10.4) |
| NVT | 20 (55.6) | 15 (71.4) | 25 (75.8) | 28 (80.0) | 88 (70.4) |
| **≥2 year of age** | **N=93** | **N=152** | **N=115** | **N=118** | **N=478** |
| PCV-7 | 20 (21.5) | 30 (19.7) | 21 (18.3) | 15 (12.7) | 86 (18.0) |
| PCV-13 | 19 (20.4) | 54 (34.9) | 32 (27.8) | 21 (17.8) | 125 (26.1) |
| NVT | 54 (58.1) | 69 (45.4) | 62 (53.9) | 82 (69.5) | 267 (55.9) |
| **Any age** | **N=129** | **N=173** | **N=148** | **N=153** | **N=603** |
| PCV-7 | 32 (24.8) | 34 (19.6) | 25 (16.9) | 19 (12.4) | 110 (18.2) |
| PCV-13 | 23 (17.8) | 55 (31.8) | 36 (24.3) | 24 (15.7) | 138 (22.9) |
| NVT | 74 (57.4) | 84 (48.6) | 87 (58.8) | 110 (71.9) | 355 (58.9) |
| ***lytA*-positive cases with Ct-values <35 (SARI program)** | | | | | |
| **<2 year of age** | **N=4** | **N=2** | **N=5** | **N=6** | **N=17** |
| PCV-7 | 0 (0.0) | 0 (0.0) | 2 (40.0) | 1 (16.7) | 3 (17.6) |
| PCV-13 | 2 (50.0) | 1 (50.0) | 2 (40.0) | 2 (33.3) | 7 (41.2) |
| NVT | 2 (50.0) | 1 (50.0) | 1 (20.0) | 3 (50.0) | 7 (41.2) |
| **≥2 year of age** | **N=22** | **N=78** | **N=44** | **N=38** | **N=182** |
| PCV-7 | 5 (22.7) | 15 (19.2) | 10 (22.7) | 7 (18.4) | 37 (20.3) |
| PCV-13 | 6 (27.3) | 43 (55.1) | 26 (59.1) | 15 (39.5) | 90 (49.5) |
| NVT | 11 (50.0) | 20 (25.6) | 8 (18.2) | 16 (42.1) | 55 (30.2) |
| **Any age** | **N=26** | **N=80** | **N=49** | **N=44** | **N=199** |
| PCV-7 | 5 (19.2) | 15 (18.7) | 12 (24.5) | 8 (18.2) | 40 (20.1) |
| PCV-13 | 8 (30.8) | 44 (55.0) | 28 (57.1) | 17 (38.6) | 97 (48.7) |
| NVT | 13 (50.0) | 21 (26.3) | 9 (18.4) | 19 (43.2) | 62 (31.1) |
| **Culture*-*positive cases (GERMS program)** | | | | | |
| **<2 year of age** | **N=84** | **N=34** | **N=39** | **N=27** | **N=184** |
| PCV-7 | 59 (70.2) | 17 (50.0) | 10 (25.6) | 5 (18.5) | 91 (49.4) |
| PCV-13 | 11 (13.1) | 7 (20.6) | 9 (23.1) | 4 (14.8) | 31 (16.8) |
| NVT | 14 (16.7) | 10 (29.4) | 20 (51.3) | 18 (66.7) | 62 (33.7) |
| **≥2 year of age** | **N=279** | **N=348** | **N=221** | **N=165** | **N=1013** |
| PCV-7 | 99 (35.5) | 132 (37.9 | 47 (21.3) | 29 (17.6) | 307 (30.3) |
| PCV-13 | 111 (39.8) | 128 (36.8) | 97 (43.9) | 58 (35.1) | 394 (38.9) |
| NVT | 69 (24.7) | 88 (25.3) | 77 (34.8) | 78 (47.3) | 312 (30.8) |
| **Any age** | **N=363** | **N=382** | **N=260** | **N=192** | **N=1197** |
| PCV-7 | 158 (43.5) | 149 (39.0) | 57 (21.9) | 34 (17.7) | 398 (33.2) |
| PCV-13 | 122 (33.6) | 135 (35.3) | 106 (40.8) | 62 (32.3) | 425 (35.5) |
| NVT | 83 (22.9) | 98 (25.6) | 97 (37.3) | 96 (50.0) | 374 (31.2) |

Abbreviations: PCV-7: 7-valent pneumococcal conjugate vaccine serotypes (included serotypes/serogroups 4, 6A/B, 9A/V/L/N, 14, 18A/B/C, 19B/F, 23F for *lyA*-positive samples and 4, 6A/B, 9V, 14, 18C, 19F, 23F for culture-positive samples); PCV-13: additional 13-valent pneumococcal conjugate vaccine serotypes (included serotypes/serogroups 1, 3, 5, 7A/F, 19A for *lyA*-positive samples and 1, 3, 5, 7F, 19A for culture-positive samples); NVT: serotypes/serogroups not included in PCV-7 or PCV-13, including samples that tested negative for the 42 serotypes detected by the serotyping assay for the *lytA*-positive cases; Ct-value: cycle threshold value.

**Figure S1:** Rates of culture-positive invasive pneumococcal disease among HIV-uninfected children <2 years of age at Chris Hani Baragwanath Academic Hospital, Soweto, South Africa, 2009-2012. **A**: Positive from blood. **B**: Positive from cerebro-spinal fluid. Seven-valent pneumococcal conjugate vaccine (PCV-7) serotypes included: 4, 6A/B, 9V, 14, 18C, 19F, 23F; additional 13-valent pneumococcal conjugate vaccine (PCV-13) serotypes included: 1, 3, 5, 7F, 19A). Non-vaccine serotypes included serotypes not included in PCV-7 or PCV-13.

**REFERENCES**

1. Carvalho M, Tondella ML, McCaustland K, et al. Evaluation of improvement of real-time PCR assays targeting *lytA*, *ply*, and *psaA* genes for detection of pneumococcal DNA. J Clin Microbiol **2007**; 45(8):2460-66. [↑](#endnote-ref-1)
2. Azzari C, Moriondo M, Indolfi G, et al. Real-time PCR is more sensitive than multiplex PCR for diagnosis and serotyping in children with culture negative pneumococcal invasive disease. PLoS One **2010**; 5(2): e9282. [↑](#endnote-ref-2)
3. Mavroidi A, Aanensen DM, Godoy D, et al. Genetic relatedness of the Streptococcus pneumoniae capsular biosynthetic loci. J Becteriol **2007**; 189(21):7841-55. [↑](#endnote-ref-3)
4. Winn WC, Allen SD, Janda WM, et al. Gram-positive cocci. Part II: streptococci, enterococci, and the “streptococcus-like” bacteria, p 672-764. In Konemans’s color atlas and textbook of diagnostic microbiology, 6th ed. **2006**; Lippincott Williams & Wilkins, Baltimore. [↑](#endnote-ref-4)
5. Austrian R. The Quellung reaction, a neglected microbiologic technique. Mt Sinai J Med **1976**; 43(6):699-709. [↑](#endnote-ref-5)
6. Whitney CG, Farley MM, Hadler J, et al. Effectiveness of seven-valent pneumococcal conjugate vaccine against invasive pneumococcal disease: a matched case-control study. Lancet **2006**; 368(9546):1495-502. [↑](#endnote-ref-6)
7. Cohen C, Moyes J, Tempia S, et al. Severe influenza-associated lower respiratory tract infection in a high HIV-Prevalence setting – South Africa, 2009-2011. Emerg Infec Dis **2013**; 19(11):1766-74. [↑](#endnote-ref-7)
8. Moyes J, Cohen C, Pretorius M, et al. Epidemiology of respiratory syncytial virus-associated acute lower respiratory tract infection hospitalizations among HIV-infected and HIV-uninfected South African children, 2010-2011. J Infect Dis **2013**; 208(S3):S217-26. [↑](#endnote-ref-8)
9. Statistics South Africa. Population statistics. <http://www.statssa.gov.za/publications/populationstats.asp>. Accessed 15 January 2015. [↑](#endnote-ref-9)
10. Actuarial Society of South Africa (ASSA). AIDS and Demographic model. <http://aids.actuarialsociety.org.za/ASSA2008-Model-3480.htm>. Accessed 15 January 2015. [↑](#endnote-ref-10)
